# Impact of China’s National Volume-Based Procurement Policy on the Affordability and Utilization of Tyrosine Kinase Inhibitors for Childhood Leukemia

**DOI:** 10.34133/hds.0453

**Published:** 2026-05-04

**Authors:** Yiran Li, Yao Xie, Fanyu Liu, Yongyi Wu, Siyu Liu, Xiao Han, Xiaohan Fan, Zhao Yang, Bin Jiang

**Affiliations:** ^1^ School of Economics, University of Chinese Academy of Social Sciences, Beijing, China.; ^2^ Pediatric Department of Peking University First Hospital, Beijing, China.; ^3^ Institute of New Structural Economics, Peking University, Beijing, China.; ^4^ School of Public Health, Kunming Medical University, Kunming, Yunnan Province, China.; ^5^ Shanxi Medical University, Jinzhong, Shanxi Province, China.; ^6^ School of Public Health, Shanghai Jiaotong University, Shanghai, China.; ^7^ Scientific Research Department, Peking University First Hospital, Beijing, China.; ^8^ Peking University First Hospital, Beijing, China.; ^9^ Research Center of Public Policy, Peking University, Beijing, China.; ^10^ Peking University School of Pharmaceutical Sciences, Beijing, China.

## Abstract

**Background:** Leukemia is the most common childhood cancer. While tyrosine kinase inhibitors (TKIs) improve survival, they impose substantial economic burdens. This study evaluates the impact of China’s National Volume-Based Procurement (NVBP) policy on the affordability and usage of TKIs in children’s hospitals. **Method:** We analyzed procurement data for imatinib, nilotinib, and dasatinib from children’s hospitals between January 2019 and December 2020. An interrupted time series model was employed to assess 3 outcomes: affordability (monthly cost-to-wage ratio), procurement volume (defined daily doses), and expenditure. Stratified analyses were conducted based on bid-winning status and generic versus originator drug types. **Results:** The NVBP policy effectively influenced some TKIs. For imatinib, which is included in the NVBP policy, imatinib procurement volume and expenditure showed significant upward trends (*P* < 0.05 and *P* < 0.01, respectively). Notably, the volume for bid-winning generics increased significantly (*P* < 0.05) upon policy implementation. The expenditure structure of TKI drugs underwent significant changes due to the policy, with a gradual increase in the proportion of expenditures for bid-winning generic drugs. **Conclusion:** The NVBP policy effectively improved TKI affordability and promoted the substitution of bid-winning generics in pediatric leukemia treatment. These changes optimized the medication expenditure structure, helping to alleviate the economic burden on families, although further policy refinements for pediatric-specific needs are warranted.

## Introduction

Leukemia is the most common malignant tumor in children; it can be divided into lymphoblastic leukemia and myeloid leukemia. Approximately 3% to 4% of pediatric acute lymphoblastic leukemia (ALL) cases turned out to be with the Philadelphia chromosome present [1]. The Philadelphia chromosome refers to the t(9;22) (q34;q11) translocation, which was first identified in patients with chronic myelogenous leukemia. This translocation leads to the formation of the BCR–ABL fusion gene [2]. The protein encoded by this gene exhibits aberrant tyrosine kinase activity, leading to uncontrolled cell proliferation and impaired apoptosis, which are key factors in the pathogenesis and progression of leukemia, commonly referred to as Philadelphia chromosome-positive acute lymphoblastic leukemia (Ph+ ALL), which lead to very poor outcome before tyrosine kinase inhibitor (TKI) emerged. Also, 95% of children with myeloid leukemia are diagnosed during the chronic phase [3]. For children in this phase, the first-line treatment is TKI, without the need for transplantation.

The introduction of TKIs has significantly improved the cure rate for Ph+ ALL and transformed treatment strategies for chronic myeloid leukemia (CML) [4]. The introduction of first-generation imatinib has significantly improved 4-year event-free survival (EFS) from 48.9% to 71.0% for CML patients [5]. Imatinib combined with intensive chemotherapy demonstrated a 3-year EFS of 88% ± 11% and a 5-year overall survival of 70% ± 6%, showing comparable long-term outcomes to human leukocyte antigen-identical sibling donor BMT [6]. Subsequently, second-generation TKIs dasatinib and nilotinib have addressed the issue of resistance to imatinib [7]. Additionally, second-generation TKIs have dual-target action by inhibiting Src/ABL, allowing for better penetration of the blood–brain barrier (BBB), making them more effective in the prevention and treatment of central nervous system leukemia. These drugs can precisely target specific molecular sites within cancer cells, inhibiting their activity and thereby preventing cancer cell growth and division.

However, the high costs of these treatments place a substantial burden on both patients and the Chinese healthcare system. In a 10-year simulation, total costs amounted to 1,020,995.35 CNY for the imatinib group and 1,035,788.50 CNY for the dasatinib group [8]. Over a lifetime, patients treated with nilotinib faced total costs of US$1,409,466. Moreover, countries with a low per-capita gross national income often experience shortages of essential cancer medications and high out-of-pocket expenses, even when these drugs are available [9].

Various strategies have been proposed to manage drug prices and enhance affordability, with centralized drug procurement and price negotiation being among the most common. The National Volume-Based Procurement (NVBP) policy represents a major reform in China’s drug procurement system. It aims to utilize market mechanisms, with the government acting as the purchaser to coordinate the drug needs of healthcare institutions. By implementing volume-based pricing through bulk purchasing, the policy seeks to lower the unit price of medications and reduce the high transaction costs associated with inefficient negotiation processes. This initiative enhances transparency in drug procurement, thereby reducing the economic pressure faced by patients.

This study attempts to clarify the impacts of the NVBP policy, including procurement and volume-based competitive bidding, on the use of TKIs in childhood leukemia. It focuses on prices, procurement volumes, and expenditures. Additionally, the research identifies factors influencing the policy’s effectiveness and examines the situations of 3 subgroups: bid-winning generics, non-bidding generics, and non-bidding originative drugs. This study provides a scientific foundation for further refining the NVBP policy, enhancing drug affordability, and alleviating patients’ medication expenses.

## Methods

### Data

The survey was carried out in 25 provinces following the methodology of the World Health Organization (WHO) and Health Action International. All the procurement quantity and total amount data of TKI drugs were obtained from the centralized drug procurement data rapid collection subsystem of the National Medical Insurance Administration. The system covers the monthly procurement records of all drugs under the name of pilot varieties and alternative varieties from 2018 to 2020, drawing on comprehensive data spanning the periods before and after the policy was enacted. The research sample is based on pediatric hospital data from 2019 to 2020 included in the aforementioned database, including 39 tertiary hospitals, 8 secondary hospitals, and 20 hospitals below the grassroots level. A children’s hospital is a general hospital focusing on the diagnosis and treatment of children’s diseases. The difference from general hospitals is that its patients are all children (basically under 18, or under 14 in some areas). Thus, the samples in this study include individual children across various provinces of China. Data regarding provincial average daily wages were retrieved from *China Statistical Yearbook 2020* and *China Statistical Yearbook 2021*.

### Drugs of interest

This study focuses on imatinib, nilotinib, and dasatinib as research subjects, based on the “L01EA: BCR-ABL tyrosine kinase inhibitors” classification in the 2022 Anatomical Therapeutic Chemical (ATC) classification guidelines [10]. Imatinib, as one of the drugs selected under the NVBP, is classified into 3 separate categories: bid-winning generic, non-bidding generic, and non-bidding originative. According to the National Medical Products Administration, an originator (innovator) drug refers to a medicinal product that is first approved for marketing domestically or internationally on the basis of complete and sufficient data on quality, safety, and efficacy, rather than relying on data from other products. Nilotinib and dasatinib, considered as alternative medications, are not included in the NVBP. Other BCR–ABL-targeted TKIs that were launched in China after January 2019 are excluded from the scope of this study.

### Measurement

This study establishes 3 outcome indicators: affordability, procurement volume, and expenditure.

Affordability: We define affordability as the ratio of monthly medication costs to the average daily wage in each province, calculated by considering drug prices and wage levels, as detailed in Eq. (1). A ratio of less than 1 indicates that the medication is considered “affordable” [11].

$$\text{Provincial affordability}=\frac{\text{Monthly drug expenditure}}{\text{Provincial daily wage}}$$ (1)

Procurement volume: We determine the defined daily doses (DDDs) for the 3 medications based on the 2022 ATC classification guidelines and the DDD allocation guidelines to assess the procurement volume of TKIs. To ensure comparability among different medications, this study employs the DDDs metric, expressed in milligrams, calculated as follows: Procurement volume (DDDs) = Monthly usage of the medication/DDD of that medication. Higher DDDs indicates a greater frequency of use for the medication.

Expenditure: Monthly expenditure for a medication is expressed in millions of Chinese yuan (CNY).

### Statistical analysis

We conducted a quasi-experimental study design using interrupted time series (ITS) to analyze the affordability, dosage, and cost of TKI drugs before and after the impact of NVBP during the study period from 2019 January 1 to 2020 December 31. Then, we used subgroup analysis by dividing imatinib into winning products and non-bidding products, as well as generic products and brand products. Lastly, we analyzed the volume and expenditure changes in these drugs.

The ITS model is shown in Eq. (2):

$$\begin{array}{c} Y_{t}=\beta_{0}+\beta_{1}\times {\text{Time}}_{t}+\beta_{2}\times {\text{Policy}}_{t}+\beta_{3}\times {\text{Trend}}_{t}+\varepsilon_{t} \end{array}$$ (2)

In the model, $Y_{t}$ denotes the monthly drug outcomes, measured by DDDs and total expenditures. ${\text{Time}}_{t}$ represents the number of months elapsed since the beginning of the observation period (January 2019); ${\text{Policy}}_{t}$ is a binary indicator capturing the implementation of the NVBP policy, coded as 0 for the pre-intervention period and 1 for the post-intervention period; ${\text{Trend}}_{t}$ is defined as 0 prior to January 2020 and equals the number of months since January 2020 in the post-policy period. The intercept term, $\beta_{0}$ reflects the baseline level of the outcome variable at *t* = 0. $\beta_{1}$ represents the estimated trend of the independent variable with respect to the unit time variable *t* before the implementation of the NVBP policy. The disturbance term $\varepsilon_{t}$ represents the random error component.

The primary parameters of interest are $\beta_{2}$ and $\beta_{3}$. Specifically, $\beta_{2}$ measured the instant change following the introduction of the NVBP policy, while $\beta_{3}$ reflected the change in the growth trend of the DDDs and expenditures after NVBP. The sum of $\beta_{1}$ and $\beta_{3}$ indicates the new growth trajectory in the post-policy period.

To quantify the policy effect, we calculated the estimated percentage change using the following procedure. First, we derived the predicted value for the final observation period. This value was obtained by summing the estimated intercept ($\beta_{0}$), the immediate post-policy level change ($\beta_{2}$), the estimate of 24 times $\beta_{1}$, and the estimate of 12 times $\beta_{3}$.The coefficients $\beta_{1}$ and $\beta_{3}$ were multiplied by the corresponding number of months to reflect the cumulative time effects before and after policy implementation. Next, we constructed counterfactual estimate for the same period under the assumption that the strategic purchasing policy had not been introduced, which is $\beta_{0}$ plus 24 times $\beta_{1}$, representing the projected outcome based solely on the pre-policy trend. Finally, the estimated percentage change was obtained by dividing the predicted value in the final period under the policy scenario by the corresponding counterfactual estimate.

To assess potential autocorrelation in the residuals, the Durbin–Watson statistic was applied. Statistical significance was determined using a 2-sided *P* value threshold of 0.05. All analyses were conducted using Stata/SE 15.1 (StataCorp).

As this research relied exclusively on aggregated secondary data and did not involve direct patient or public participation, ethical approval and informed consent were not required.

## Results

### Affordability

**Fig. 1.**
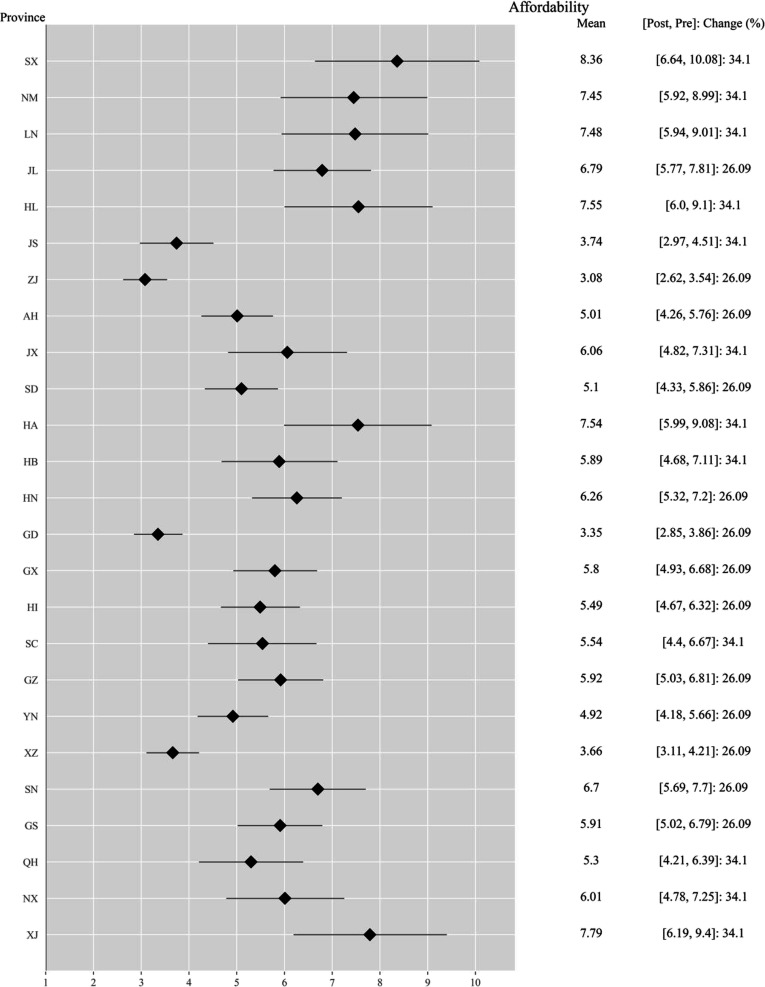
Forest plot of imatinib affordability by province.

The implementation of the NVBP policy has significantly improved the affordability of imatinib (Fig. 1). Before the policy was enacted, affordability varied by province, ranging from 3.5 to 10.1, with an average of 6.9. After implementation, affordability dropped to a range of 2.6 to 6.6, with some provinces experiencing reductions as high as 34.1%. The average affordability across provinces following the intervention was 4.8. Economically developed coastal regions demonstrated the best affordability, such as Zhejiang (3.08), Guangdong (3.35), and Jiangsu (3.74). In contrast, inland and northeastern provinces faced higher burden ratios, with Shanxi (8.36), Xinjiang (7.79), Heilongjiang (7.55), and Henan (7.54) recording the highest values. This distribution indicates that while drug prices decreased, the affordability gap between eastern coastal areas and western/northeastern regions remains prominent. Overall, the NVBP policy has effectively lowered drug prices, substantially reducing the financial burden of medication costs for patients.

### Procurement volume

**Fig. 2.**
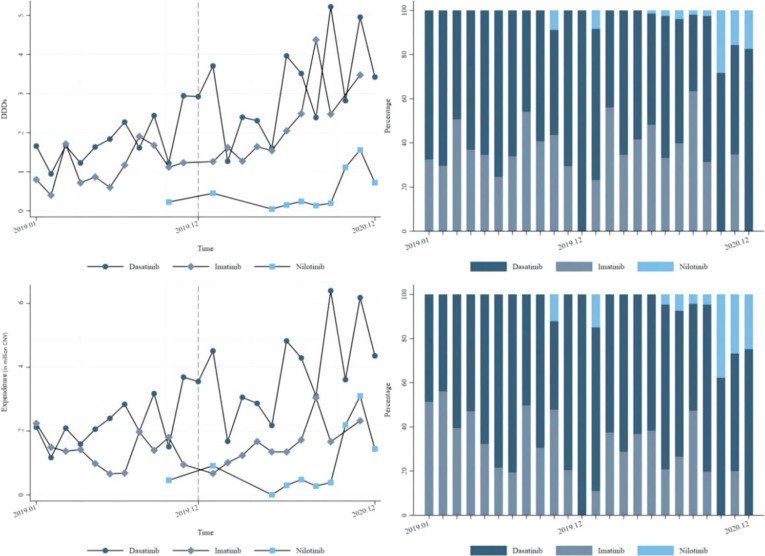
Procurement volume (defined daily doses [DDDs]) and expenditure of antileukemics at the national level.

This study begins by analyzing the monthly changes in DDDs for the 3 TKIs at the national level (Table 1 and Fig. 2). For imatinib, which is included in the NVBP, there is a significant upward trend influenced by the NVBP policy (*P* < 0.05), although no significant increase in DDDs was observed after the month of policy implemented (*P* > 0.05). In contrast, for nilotinib and dasatinib, the effects of the NVBP policy are not significant (*P* > 0.05), showing no notable instantaneous or trend changes before and after the policy.

**Table 1.** Procurement volume (DDDs) and expenditure of antileukemics at the national level (ITS). Standard errors in parentheses. Coefficients reported as 0 with missing standard errors (.) indicate that the corresponding variable was omitted from the model estimation due to collinearity or lack of variation, implying that the change was statistically negligible.

Model Estimates	Procurement volume (in thousand DDDs)			Expenditure (in million CNY)		
	Dasatinib	Imatinib	Nilotinib	Dasatinib	Imatinib	Nilotinib
Pre-intervention slope	0.102	0.0691	0.0835	0.0128	−0.00394	0.0167
	(0.0832)	(0.0531)	(0.0487)	(0.00982)	(0.00469)	(0.00974)
Change in slope	0.0405	0.185*	0	0.00566	0.0202**	0
	(0.105)	(0.0786)	(.)	(0.0124)	(0.00694)	(.)
Change in intercept	−0.165	−0.913	−0.521	−0.0272	−0.0633	−0.106
	(0.711)	(0.525)	(0.681)	(0.0839)	(0.0464)	(0.136)
Constant	1.155	0.694	−0.610	0.147*	0.160***	−0.121
	(0.564)	(0.360)	(0.675)	(0.0666)	(0.0318)	(0.135)

DDDs, defined daily doses; ITS, interrupted time series; CNY, Chinese yuan

*P < 0.05, **P < 0.01, ***P < 0.001.

**Fig. 3.**
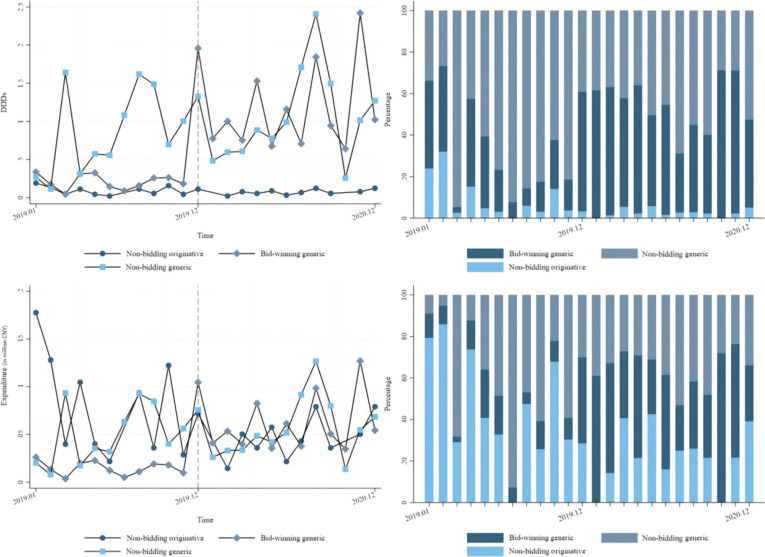
Procurement volume (defined daily doses [DDDs]) and expenditure of specific categories of imatinib at the national level.

For imatinib, we analyzed the monthly changes in DDDs at the national level across 3 subgroups: bid-winning generic, non-bidding generic, and non-bidding originative (Table 2 and Fig. 3). In the early stage of the study, the market shares of non-bidding generic and bid-winning generic drugs were comparable, while the proportion of non-bidding originative drugs gradually declined. After the implementation of the policy, bid-winning generics began to show a competitive market advantage. Prior to the NVBP, the DDDs for bid-winning generics were also at a relatively low level. Following the introduction of the policy, the DDDs for bid-winning generics showed a significant increase in the month of policy implementation (*P* < 0.05).

**Table 2.** Procurement volume and expenditure of specific categories of imatinib (ITS). Standard errors in parentheses.

	Procurement volume (in thousand DDDs)			Expenditure (in million CNY)		
Specific categories of imatinib	Non-bidding originative	Bid-winning generic	Non-bidding generic	Non-bidding originative	Bid-winning generic	Non-bidding generic
Pre-intervention slope	−0.00514	−0.00288	0.0786	−0.00700	−0.000471	0.00418
	(0.00450)	(0.0426)	(0.0527)	(0.00365)	(0.00228)	(0.00287)
Change in slope	0.00755	0.0161	−0.0369	0.00854	0.00113	−0.00216
	(0.00599)	(0.0539)	(0.0667)	(0.00486)	(0.00288)	(0.00364)
Change in intercept	−0.00639	0.900*	−0.472	−0.00545	0.0463*	−0.0270
	(0.0416)	(0.364)	(0.450)	(0.0338)	(0.0195)	(0.0245)
Constant	0.123***	0.226	0.378	0.120***	0.0174	0.0242
	(0.0304)	(0.289)	(0.357)	(0.0247)	(0.0154)	(0.0195)

DDDs, defined daily doses; ITS, interrupted time series; CNY, Chinese yuan

*P < 0.05, **P < 0.01, ***P < 0.001.

### Expenditures

We then analyzed the changes that happened in expenditure (Table 1 and Fig. 2). As shown in Fig. 2, the expenditures for dasatinib and imatinib far exceeded that of nilotinib. Imatinib was significantly affected by the policy, showing a notable upward trend in expenditure (*P* < 0.01).

We categorized imatinib in the same way as in the DDD analysis, examining the monthly changes in expenditure for the 3 subgroups: bid-winning generic, non-bidding generic, and non-bidding originative (Table 2 and Fig. 3). Table 2 indicates that the NVBP did not produce a notable effect on the expenditure of non-bidding drugs (*P* > 0.05), whereas bid-winning generics experienced an increase in expenditure of 46,300 CNY in the month of policy implementation (*P* < 0.05).

Figure 3 reveals that for originator drugs, expenditure exhibited a fluctuating downward trend over time. This is partly due to the continuous decline in prices following the expiration of patents and partly due to the increasing impact of the lower cost of high-quality generics. Given the high prices of originator drugs, they consistently comprised a large portion of TKI expenditure; however, this phenomenon changed significantly after the NVBP, with the expenditure share of affordable and high-quality generics increasing substantially. Expenditures for bid-winning generics remained low prior to the NVBP but experienced a sharp rise following policy implementation, after which they fluctuated. There was no significant change in expenditure for non-bidding generics before and after the NVBP.

## Discussion

This study employed the ITS method to evaluate the impact of the NVBP policy on the affordability, procurement volume (DDDs), and expenditures of 3 TKIs used in the treatment of childhood leukemia in China from January 2019 to December 2020. Our research indicates that the NVBP policy significantly lowered drug prices, markedly improved the affordability of TKIs, and facilitated the use of generics, thereby contributing to an improvement in patients’ economic conditions.

Introduced in China in the early 2000s, the originator imatinib (Gleevec) was priced far beyond average income levels, imposing a substantial financial burden on patients. Although its price was reduced and it was later included in major disease insurance schemes in some provinces, affordability remained a major concern. Following the introduction of the NVBP policy, the prices of TKIs saw a significant reduction. Research by Yuan et al. [12] indicated that the average affordability of the first batch of bid-winning drugs improved from 8.2 days’ wages prior to the policy implementation to 2.8 days’ wages afterward, indicating that the NVBP greatly enhanced drug affordability, which aligns with the findings of this study. Within 3 years of the NVBP implementation, over 260 billion CNY (approximately US$36.3 billion) has been saved, alleviating the economic pressure on patients [13].

This study found that prior to the adoption of the NVBP policy, the DDDs for imatinib were similar to those for dasatinib, while the DDDs for nilotinib were the lowest. Following the NVBP implementation, the DDDs for dasatinib exhibited a fluctuating trend but were not significantly affected by the policy (*P* > 0.05). Similarly, the adoption of the NVBP policy had little influence on the changes in DDDs for nilotinib (*P* > 0.05). In contrast, imatinib showed a notable upward trend in DDDs, particularly influenced by the policy (*P* < 0.05). In the analysis of imatinib, we differentiated among various types of drugs and found that the DDDs for bid-winning generics significantly increased in the month of implementation. This finding is consistent with the research conducted by Liu et al. [14]. However, the NVBP policy did not notably change the expenditure levels of non-bidding generics and originator drugs.

Regarding the expenditure on TKIs, imatinib showed a significant upward trend in expenditure following the implementation of the policy (*P* < 0.01), although there was no notable impact during the month of policy implementation. Notably, the expenditure for bid-winning generics experienced a significant increase in the month of policy implementation (*P* < 0.05).

In summary, the NVBP policy had no significant impact on dasatinib and nilotinib, while it exhibited a notable upward trend for imatinib. The policy also had a significant instantaneous effect on bid-winning generics in the month of implementation. Following the policy implementation, bid-winning generics were significantly affected in that same month. The expenditure for non-bidding originative showed little change and was not significantly influenced by the policy. This resilience likely stems from strong family values in China, where parents prioritize treatment quality over cost. Influenced by the belief that “expensive implies superior”, many parents prefer imported originators, harboring doubts about domestic generics. Additionally, a reluctance to switch medications for stable pediatric patients contributes to the sustained demand for non-bidding drugs.

Previous studies on adults have shown that the NVBP policy brought about a pronounced increase in usage and market share for bid-winning imatinib, greatly reducing the medication burden and related expenditures [15]. The health issues of children and adolescents are receiving increasing attention [16]. Our study conducted in children’s hospitals found different results. While we also observed substantial reductions in drug prices and improvements in affordability, the magnitude of changes in utilization was relatively smaller compared with adult patients. This difference likely reflects the unique characteristics of pediatric care: children often require shorter or more variable treatment courses, prescribing decisions are more conservative due to heightened safety concerns, and parental preferences strongly influence medication choice. These findings highlight that although NVBP policies are broadly effective across populations, the mechanisms and intensity of impact differ between adults and children, requiring policy designed for different ages.

One reason for this discrepancy is the strong family values in China, where children are often viewed as the center of the family and the hope for the future. As children are under parental guardianship, their medication usage is heavily influenced by parental decisions. Parents can be seen as key determinants that either maximize positive clinical outcomes or lead to significant health setbacks [17]. Parents strive to provide the best for their children and are often willing to invest significantly in their treatment when health issues arise [18]. Therefore, even though the NVBP policy significantly lowered the prices of TKIs, the effect of the policy on the treatment costs for childhood leukemia is not significant. Additionally, when faced with their children’s health issues, parents tend to be more cautious. Influenced by traditional medical beliefs, they are more likely to incur higher costs to seek care at renowned hospitals with rich medical resources, believing that these hospitals possess more advanced equipment and more specialized physicians, which can lead to better treatment outcomes. Many parents also adhere to the belief that “expensive means good”, leading them to favor higher-priced medications. Additionally, some choose to purchase medications and health supplements from overseas based on recommendations or trust in product quality. Some parents harbor biases regarding the effectiveness and safety of imported drugs, believing they are superior to domestic generics, leading to lower trust in the efficacy of domestic products. As a result, they tend to prefer higher-priced imported medications over the bid-winning domestic generics that benefit from the NVBP policy.

Another factor contributing to these findings is the difference in TKI indications between children and adults. In the pediatric population, TKIs are primarily used for Ph+ ALL, which involves distinct treatment protocols characterized by shorter durations and lower dosages. This typically results in a lower overall medication burden for children, further limiting the NVBP policy’s effect on their drug expenditures.

Health inequities have been witnessed around the world [19,20]. As the world’s second most populous country, China is increasingly facing a mismatch between the growing demand for pediatric medications and the availability of medical resources, highlighting inadequacies in medical policies concerning children’s medications. The characteristics of the NVBP policy, which focuses on bulk procurement and price negotiation based on volume, often result in the selection of mainstream specifications and dosage forms that cater to larger demands, leaving pediatric formulations and specifications frequently unselected. Additionally, the labeling information for bid-winning medications often lacks clear or standardized guidelines for pediatric use, complicating hospitals’ efforts to administer these medications to children. In China, Guangdong Province has implemented measures to better address pediatric medication needs within the NVBP policy framework. On 2022 January 19, the Guangdong Provincial Drug Trading Center issued the “Consolidated Procurement Document for Drugs Such as Diphenhydramine for the Guangdong Alliance”, indicating that drugs intended exclusively for pediatric use will be categorized based on the actual registering companies and products, rather than solely on price. This approach involves a comprehensive assessment of the pricing levels and supply assurance capabilities of companies providing pediatric-specific medications to ensure that such drugs can secure bid-winning status under the bulk procurement policy.

Currently, China has implemented various measures to reduce the economic burden of childhood leukemia on families. The country is enhancing the coverage of medical insurance policies to increase the reimbursement rates for the treatment of childhood leukemia, thereby improving lowering costs, treatment outcomes, lowering costs, and alleviating the financial strain on patients’ families. Additionally, the government actively encourages and supports social participation in the treatment of childhood leukemia through fundraising, charitable foundations, and other initiatives to gather funds and provide financial assistance to impoverished children to further reduce the economic burden on their families.

Based on this research, we can conclude that the NVBP policy has significantly impacted the TKIs required for treating childhood leukemia, improved the affordability of medications, promoted equity in medical services, and alleviated the economic burden on patients. This policy not only decreased the prices of bid-winning medications but also affected the pricing and usage of non-bidding alternatives within the same therapeutic area. Additionally, the NVBP has refined China’s pharmaceutical procurement system by reducing transaction costs and enhancing the efficiency and fairness of drug purchasing. However, our study indicates that further steps can be taken to enhance the NVBP policy regarding pediatric medications. While it effectively secures low prices based on demand volume, it is vital to ensure that the specific needs of various medication subcategories are addressed and that medication safety is maintained.

This study uses DDDs to measure the procurement volume of TKIs. It should be noted that the DDD, as defined by the WHO Collaborating Centre for Drug Statistics Methodology, defined by the WHO Collaborating Centre for Drug Statistics Methodology, is generally based on adult use. For medications approved for pediatric use, recommended doses vary according to age and body weight. In the present study, DDDs were employed as a standardized procurement-based metric to assess relative changes in drug utilization over time and to ensure comparability across drugs and study periods at the aggregate hospital level. The WHO has emphasized that, despite their limitations, DDDs remain suitable for monitoring trends in drug use when patient-level dosing information is unavailable.

Moreover, our ITS analysis focuses on within-drug changes before and after the implementation of the NVBP policy. Any systematic bias arising from the use of adult-based DDD definitions is therefore expected to remain relatively stable over time and is unlikely to materially affect the estimated policy-related changes.

Nevertheless, DDD measurement may underestimate or overestimate true pediatric medication exposure and cannot capture age- or weight-specific dosing heterogeneity. Accordingly, the findings should be interpreted as reflecting changes in procurement patterns rather than direct measures of pediatric clinical dosing. Future studies using individual patient prescription data or pediatric-specific dosing indicators would be valuable to further validate these results.

Finally, it is important to recognize certain limitations of this study. Firstly, affected by data availability, the research period is limited to 2 years (2019 to 2020). Consequently, this study could not assess the long-term sustainability or lagged impacts of the NVBP policy. Furthermore, the study period (2019 to 2020) coincides with the outbreak of COVID-19. Although the study period includes the COVID-19 pandemic, our research is based on institutional procurement data rather than individual patient consumption records. As a result, the findings are less likely to be affected by pandemic-related restrictions, such as patients’ ability to visit hospitals to obtain medications.

## Conclusion

This study evaluated the impact of the NVBP policy on the treatment medications for childhood leukemia. Our results indicate that the NVBP policy significantly increased the usage of bid-winning generics. Although the absolute procurement volume of non-bidding originators remained relatively stable rather than decreasing sharply, the relative market share of bid-winning generics expanded substantially. The NVBP has introduced high-quality medications at more affordable prices, significantly improving the affordability and accessibility of TKIs and enhancing the treatment conditions for children with leukemia. Therefore, refining the NVBP policy is of great significance. Future pharmaceutical procurement should include differentiated mechanisms for drugs indicated solely for pediatric use, thereby maximizing the positive impact of the procurement policy on children’s medications.

## Data Availability

The data that support the findings of this study are available from the China Health Insurance Bureau, which were used under license for the current study and so are not publicly available but are available from the corresponding author on reasonable request.
